# Herbivores with similar feeding modes interact through the induction of different plant responses

**DOI:** 10.1007/s00442-015-3344-0

**Published:** 2015-05-30

**Authors:** Elisa F. de Oliveira, Angelo Pallini, Arne Janssen

**Affiliations:** Department of Entomology, Federal University of Viçosa, Viçosa, Minas Gerais Brazil; Institute for Biodiversity and Ecosystem Dynamics, University of Amsterdam, Science Park 904, 1098 XH Amsterdam, The Netherlands

**Keywords:** Induced defense, Herbivory, Performance, Proteinase inhibitors, Specificity of plant responses

## Abstract

Plants respond to attacks by herbivores with various defences, which are mounted through the activation of different biochemical pathways that are known to interact. Thus, the attack of a plant by one herbivore species may result in changes in the performances of other species on the same plant. It has been suggested that species with comparable feeding modes induce similar plant defences and such herbivores are therefore expected to have a negative effect on each other’s performance. We studied two closely related phytophagous mite species with identical feeding modes. Yet, one of the species (*Tetranychus urticae*) induces tomato plant defences, whereas the other (*T. evansi*) reduces them. We found that the “inducing” species benefits from the downregulation of defences by the “reducing” species, which, in turn, suffers from the induction of defences by the inducing species. Moreover, the performances of the two mite species on leaves that were previously attacked by both species simultaneously were intermediate between that on leaves previously attacked by each of the mites separately. The activity of proteinase inhibitor, a defensive compound, was not found to be intermediate in leaves attacked by both species simultaneously—it was almost as high as the activity seen in leaves with defences induced by *T. urticae*. Oviposition rates of *T. urticae* showed a nonlinear correlation with inhibitor activity, suggesting that it is potentially problematic to use this activity as an indicator of the level of plant defence. Our results show that herbivores with similar feeding modes have opposite effects on plant defence and differentially affect each other’s performance on co-infested plants.

## Introduction

Plants employ a wide range of induced defences in response to herbivore attack. These defences result in morphological changes and synthesis of secondary metabolites, which cause a decrease in herbivore performance (Karban and Baldwin 1997; Walling 2000; Howe and Jander 2008; Alba et al. 2011) or enhance the performance of natural enemies of the herbivores (Turlings et al. 1995; Sabelis et al. 1999; Rasmann et al. 2005; Dicke and Baldwin 2010; Abe et al. 2012). The induction of these plant defences depends on the ability of the plant to identify and recognize its attackers (Baldwin and Preston 1999; Walling 2000; de Vos et al. 2005; Wu and Baldwin 2009), and varies with the herbivore species (Stout et al. 1998; de Vos et al. 2005; Rodriguez-Saona et al. 2010) and time since attack (Kant et al. 2004). Different herbivore species on the same plant can thus affect each other through the defences that they induce (Viswanathan et al. 2005; Kessler and Halitschke 2007; Kaplan et al. 2009). When the herbivores affect each other negatively, this interaction is sometimes misleadingly referred to as “indirect competition” to distinguish it from resource competition, which is, however, also an indirect interaction.

Attacks by one herbivore species may reduce or increase plant defence against other herbivore species (Karban and Carey 1984; Karban and Baldwin 1997; Viswanathan et al. 2005; Rodriguez-Saona et al. 2005; Poelman et al. 2008; Bruessow et al. 2010). It has been suggested that species with comparable feeding modes induce similar plant defences and they are therefore expected to have a negative effect on each other’s performance (Rodriguez-Saona et al. 2005; Howe and Jander 2008; Soler et al. 2012). Indeed, early studies of plant defences showed that two closely related mite species induced resistance with similar effects on the performance of one of these species (Karban and Carey 1984). To date, studies on the effects of simultaneous plant attacks by various herbivore species have mainly focused on herbivores of different feeding guilds, which are thought to induce different defensive pathways (Rodriguez-Saona et al. 2005, 2010; Howe and Jander 2008; Soler et al. 2012). We investigated the effects of simultaneous attacks of tomato plants by two herbivores with similar feeding modes, but with opposite effects on plant defence responses.

The spider mite *Tetranychus urticae* is well known for inducing defences in various plant species, including tomato (Li et al. 2002; Kant et al. 2004, 2008; Ament et al. 2004), although there is substantial variation in induction among strains, with some strains even suppressing plant defences (Kant et al. 2004, 2008; Alba et al. 2015). It feeds on plant tissue by piercing parenchyma cells and sucking out their contents. This feeding induces direct plant defences such as proteinase inhibitor activity within one day (Kant et al. 2004). Earlier feeding by defence-inducing *T. urticae* results in lower performance of later-arriving herbivores (Karban and Carey 1984; Karban et al. 1987; Sarmento et al. 2011a). Although *T. evansi* has the same feeding mode, it performed better on plants that had previously been attacked by conspecifics (Sarmento et al. 2011a). This increased performance coincided with reduced levels of defence-related plant constituents such as proteinase inhibitors, which were below the levels in plants that had not been attacked (Sarmento et al. 2011a). These inhibitors hamper the action of digestive proteinases present in the herbivore gut (Ryan 1990; Koiwa et al. 1997), including those of spider mites (Li et al. 2002; Kant et al. 2004), and are normally induced by herbivore attacks. The low activity of proteinase inhibitors in leaves previously attacked by *T. evansi* coincided with a lack of upregulation of the proteinase inhibitor gene *WIPI*-*II*, which is dependent on the jasmonic acid pathway. *PR*-*P6*, a marker gene of the salicylic acid pathway, was also not upregulated by attacks from *T. evansi*, suggesting that the lower defence in plants that had previously been attacked by *T. evansi* was not caused by negative crosstalk between the two pathways. This was recently confirmed using several marker genes for both pathways (Alba et al. 2015). The reduction of defences in tomato plants by *T. evansi* also resulted in better performance of *T. urticae* (Sarmento et al. 2011a, b). It is not known yet how simultaneous attacks by these two herbivores affect tomato plant defence. Here, we investigated the effect of simultaneous attacks of the same leaf by both spider mite species on locally induced plant defences.

Besides *T. evansi*, there are several other examples of herbivores that interfere with plant defence responses (Musser et al. 2002; Bede et al. 2006; Lawrence et al. 2008). However, most of these studies did not quantify the effects of defence suppression on insect performance, leaving open the possibility that defence suppression could benefit the natural enemies of the herbivores and thus the plant (Kahl et al. 2000). Several recent studies have specifically shown effects of defence suppression on herbivore performance (Kant et al. 2008; Sarmento et al. 2011a, b; Consales et al. 2012; Dafoe et al. 2013; Alba et al. 2015). Here, we use a similar approach and quantify plant defences through herbivore performance (oviposition) and by measuring the activity levels of proteinase inhibitors in plant tissue to investigate the effect of simultaneous attack by “inducer” (i.e., *T. urticae*) and “reducer” (i.e., *T. evansi*) herbivores on plant defence.

To evaluate the effects of simultaneous attacks on plant defence, it is essential to know the timing of plant responses to herbivore attacks. Whereas it is known that *T. urticae* induces direct plant defences in tomato within 1 day, there is little information on the timing of the effects of *T. evansi* on plant defences. Sarmento et al. (2011a) found increased oviposition of *T. evansi* on tomato plants 7 days after attack by conspecific mites, but it is possible that a shorter period of attack results in similar downregulation of plant defences. Therefore, we compared the timing of the reduction of plant defences by *T. evansi* with the timing of induction by *T. urticae* to subsequently investigate the effects of simultaneous attacks.

## Materials and methods

### Plant material

Tomato seeds (*Solanum lycopersicum* var Santa Clara I-5300) were sown in a commercial plant substrate (Bioplant^®^, Bioplant Misturadora Agrícola LTDA) in a polystyrene tray (8 × 16 cells), and kept inside a fine-meshed cage in a greenhouse to avoid infestation with herbivores. After 21 days, the plants were transferred to plastic pots (2 L) that contained a mixture of soil plus bovine manure (3:1) and fertilizer (4–14–8 N–P–K). Tomato plants were further grown in mite-proof screen cages in a greenhouse until they were 45 days old and had at least four completely developed leaves. Subsequently, they were used either for the experiments or for spider mite rearing.

### Mites

*Tetranychus evansi* and *T. urticae* were obtained in 2002 from naturally infested tomato plants of the same variety as above in a greenhouse at the Federal University of Viçosa, Brazil (Sarmento et al. 2011a). Both species were cultured on detached tomato leaves, with the petiole inserted into a PVC tube containing water to maintain leaf turgor. Tubes with infested leaves were kept in PVC trays filled with detergent and water (1:25, v/v), which served to prevent the escape of mites and invasions by mites and other non-flying arthropods. The mass culture was maintained in a room at 25 ± 3 °C and 70–90 % relative humidity and 12 h of light per day.

### Timing of induction of direct plant defences

The third leaf down of randomly selected tomato plants that were 45 days old (four fully developed leaves) was infested for 0 (no infestation, control), 1, 2, 3 or 4 days with 100 adult females of *T. urticae* or *T. evansi*, while the other leaves were kept clean. Four plants were used for each treatment, so a total of 40 plants were used for this experiment. Insect glue (Cola Entomológica; Bio-Controle, São Paulo, Brazil) was applied to the petioles of leaves on which mites were released to prevent them from moving to another leaf. Leaves of control plants from the same batch that were the same age were also treated with glue. Plants were kept inside mite-proof screen cages in a greenhouse during the experiment. After infestation for 1–4 days, 20 leaf discs (∅ 12 mm) were made per plant from all leaflets of the leaves damaged by *T. evansi* or *T. urticae* and from corresponding leaves of uninfested control plants using a cork borer (Huffaker et al. 1970). The mites as well as their web and eggs were carefully removed from the discs with a fine brush under a stereoscopic microscope, taking care not to damage the leaf discs any further. Discs were subsequently kept in Petri dishes (Ø 8 cm) containing wet cotton wool. Two leaflets of the same leaf were used to assess proteinase activity (see below).

We used oviposition rates of *T. evansi* and *T. urticae* as stand-in measures of herbivore performance. The oviposition rate of spider mites is closely correlated to the population growth rate (Sabelis 1991; Janssen and Sabelis 1992). The oviposition rates of *T. evansi* and *T. urticae* were measured on the discs. Because the oviposition rate of spider mites decreases with age (Sabelis 1991), female mites of a similar age were used in the oviposition experiments. To obtain such cohorts, several adult females were allowed to lay eggs on detached tomato leaves on wet cotton wool. The adults were removed after 24 h and the eggs were reared to adulthood. One randomly selected adult female of *T. evansi* or *T. urticae* was placed on each disc 2 days after it had turned adult. The oviposition rate was measured after 4 days (28 ± 2 °C; 70 ± 10 % RH 12 h light). Oviposition rates were averaged per spider mite species and per plant. Spider mites that did not survive the entire period of oviposition were discarded (the average per plant therefore consisted of the average of up to ten mites of each species). The experiments with plants infested by *T. evansi* and *T. urticae* could not be carried out at the same time for logistical reasons. Treatments can therefore only be compared to controls within the same experiment.

### Simultaneous attack by *T. evansi* and *T. urticae*

A preliminary experiment was performed to investigate the effect of different numbers of mites damaging the plants on the subsequent performance of both mite species. The third leaf of randomly selected tomato plants was infested with either 100 or 200 adult females of either species for 4 days, and leaf discs were made from the infested leaves as above. Four plants were used per treatment, i.e., 16 in total. Subsequently, the oviposition rates of individual females of both species (ten females of each per plant) were assessed after 4 days as described above.

To study the effects of simultaneous attack, plants were either concurrently infested with adult females of *T. evansi* and *T. urticae*, with either of the two species separately, or they were not infested. Based on the results of the preliminary experiment, we decided to infest plants with 100 adult mites of each species in the case of co-infested plants (200 mites in total), whereas plants with only one of the two species received 100 mites. The third leaf from below of randomly selected 45-day-old tomato plants was infested for 1 day as described above while the other leaves were kept clean. There were four plants per treatment; 16 plants in total. Subsequently, the damaged leaves were cleaned and leaf discs were made as above. One adult female of *T. evansi* or *T. urticae* was released per leaf disc as above and the oviposition rate was evaluated after 4 days.

### Proteinase inhibitor assays

The proteinase inhibitor (PI) activity was measured in the same leaves as used for the oviposition experiments. Two leaflets per infested leaf (above) and from the corresponding control leaf were frozen in liquid nitrogen and stored at −80 °C. Subsequently, each sample was ground with mortar and pestle and a crude protein extract was obtained as described by Otha et al. (1986). Essentially, the leaves were homogenized in extraction buffer (0.1 M Tris–HCl buffer, pH 8.2 and 20 mM CaCl_2_; 1:3 w/v); the homogenate was then centrifuged at 17,200×*g* for 30 min at 4 °C and the supernatant was collected, which was used to determine the protein content and all other assays. Protein concentration was determined by the method described by Bradford (1976), using a solution of 0.2 mg/ml bovine serum albumin as standard. A standard spectrophotometric assay was used to measure trypsin inhibitory activity in the supernatant. A 100-μL aliquot of trypsin (4.7 × 10^−5^ M) was mixed with 100 μL of the supernatant and 500 μL extraction buffer (0.1 M Tris–HCl buffer, pH 8.2 and 20 mM CaCl_2_). The mixture was incubated at room temperature for 5 min. Controls consisted of 600 μL extraction buffer and 100 μL trypsin (4.7 × 10^−5^ M). A 700-μL aliquot of the mixture (tests and controls) was added to 500 μL extraction buffer and 500 μL Na-benzoyl-d,l-arginine-4-nitroanilide hydrochloride (d,l-BApNA, 1.2 mM). Trypsin activity was monitored for 150 s at intervals of 30 s at 410 nm absorbance on a spectrophotometer. The difference between the absorbances measured at 150 s and 60 s was used to determine the trypsin activity. Measurements were performed in triplicate per sample. The results obtained were converted to milligrams trypsin inhibited per gram of protein according to the following equation: mg trypsin inhibited per gram of protein = *AB*/1000*PC*, where *A* = enzyme control − absorbance at 410 nm of the extract, *B* = sample dilution, *P* = protein concentration of the extracts (g/mL), and *C* = trypsin factor, the result of the activity of 1 μg of trypsin on the substrate d,l-BApNA measured at 410 nm; for the combination of trypsin and d,l-BApNA, the result is 0.019 (Kakade et al. 1974).

### Statistics

Differences in mean oviposition rates per plant among treatments were tested with a generalized linear model (GLM) with a Gaussian error distribution (R Development Core Team 2013). Contrasts among treatments were assessed by aggregating non-significant treatment levels in an a posteriori stepwise procedure (Crawley 2007). Differences in PI activity were analyzed with a GLM with a Gaussian error distribution.

We correlated the oviposition rates of the mites with PI activity measured in the same leaf. Because we expected oviposition not to depend linearly on proteinase inhibitor activity but to follow a dose–response curve, we also fitted such a curve (a three-parameter logistic model) of the form

$${\text{Ovip}} = a + \frac{b}{{1 + {\text{e}}^{{c - {\text{PI}}}} }},$$

where Ovip = oviposition rate of the spider mites, PI = the proteinase activity, and *a*, *b*, and *c* are parameters that were estimated with the nls function in R (R Development Core Team 2013). Models were compared with the “anova” function in R (R Development Core Team 2013) and with Akaike’s information criterion (AIC), and nonsignificant parameters were removed from the model. We also used piecewise regression to identify the correlation of oviposition rate within various ranges of proteinase inhibitor activity and to assess the approximate value of the inflection point of the dose–response curve. In short, piecewise regression consists of fitting different linear regressions to various ranges of the data, choosing the ranges that result in the lowest residual standard error (Crawley 2007).

## Results

### Timing of induction of direct plant defences

**Fig. 1:**
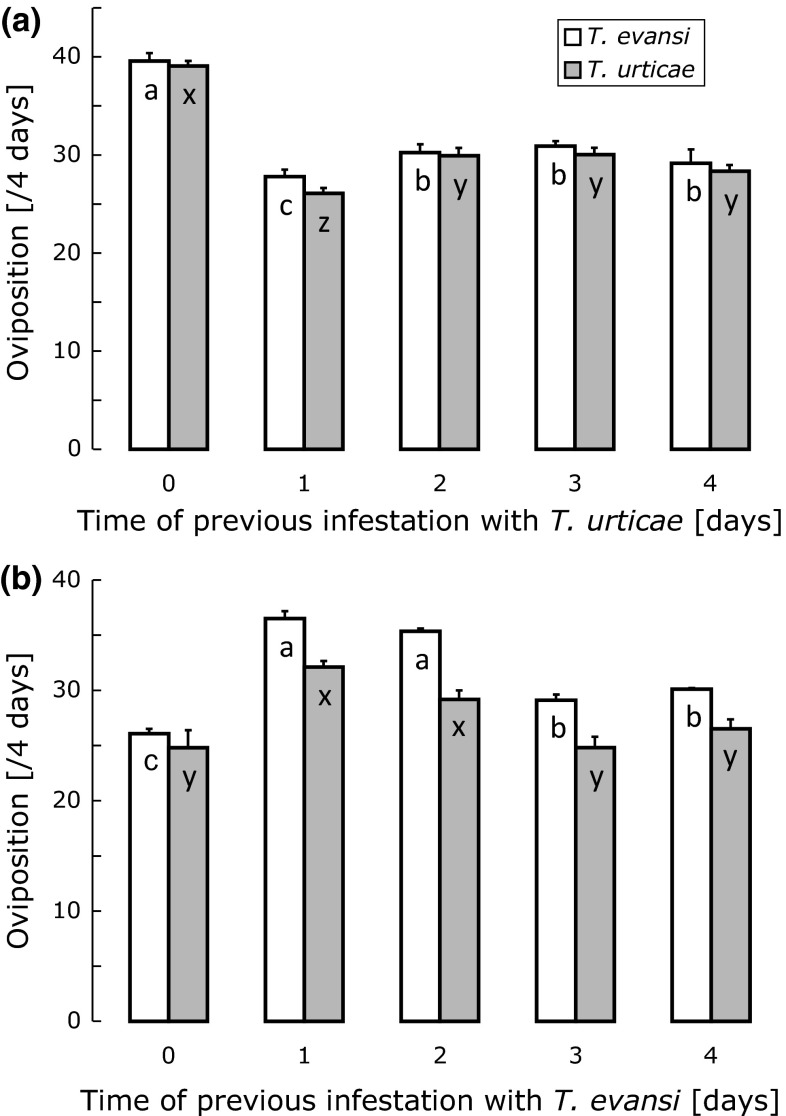
Average oviposition rates (eggs/female/4 days + SE) of *Tetranychus evansi* (*white bars*) and *T. urticae* (*gray bars*) on discs made from leaves that were previously attacked by *T. urticae* (**a**) or *T. evansi* (**b**) for 1–4 days or that were not previously attacked (0 days). Oviposition rates were averaged over a maximum of 10 adult females per plant, and each treatment was repeated on 4 plants. For each panel and each species,* bars with the same letter* are not significantly different (contrast among treatments after a GLM). For logistical reasons, the experiments corresponding to panels **a** and **b** were not carried out at the same time. Therefore, treatments can only be compared within the same experiment

The oviposition rates of *T. urticae* and *T. evansi* were significantly affected by previous attack of the plants by *T. urticae* (GLM, *T. urticae*: *F*_4,15_ = 57.4, *P* < 0.0001; *T. evansi*: *F*_4,15_ = 25.7, *P* < 0.0001). Oviposition on leaves of clean plants (0 days of previous infestation) was significantly higher than on leaves previously attacked by *T. urticae*, and the oviposition after 1 day of previous infestation by *T. urticae* was lower than that after several days of previous infestation (Fig. 1a). These data confirm that *T. urticae* induces direct plant defences in tomato plants within 1 day (Kant et al. 2004).

The oviposition rates of both species was also significantly affected by previous attacks by *T. evansi* (GLM, *T. urticae*: *F*_4,15_ = 9.5, *P* = 0.0005; *T. evansi*: *F*_4,15_ = 95.5, *P* < 0.0001) (Fig. 1b). Oviposition on leaves that were previously attacked by *T. evansi* for 1 or 2 days was significantly higher than that on leaves of clean plants (0 days of previous infestation) (Fig. 1b). Oviposition by *T. evansi* on plants that were previously attacked by *T. evansi* for 3 and 4 days was lower than that on plants attacked for 1 or 2 days, but higher than that on clean plants (Fig. 1b). Oviposition by *T. urticae* on plants previously attacked by *T. evansi* for 3 and 4 days was not significantly higher than on plants that were not attacked before (Fig. 1b).

**Fig. 2:**
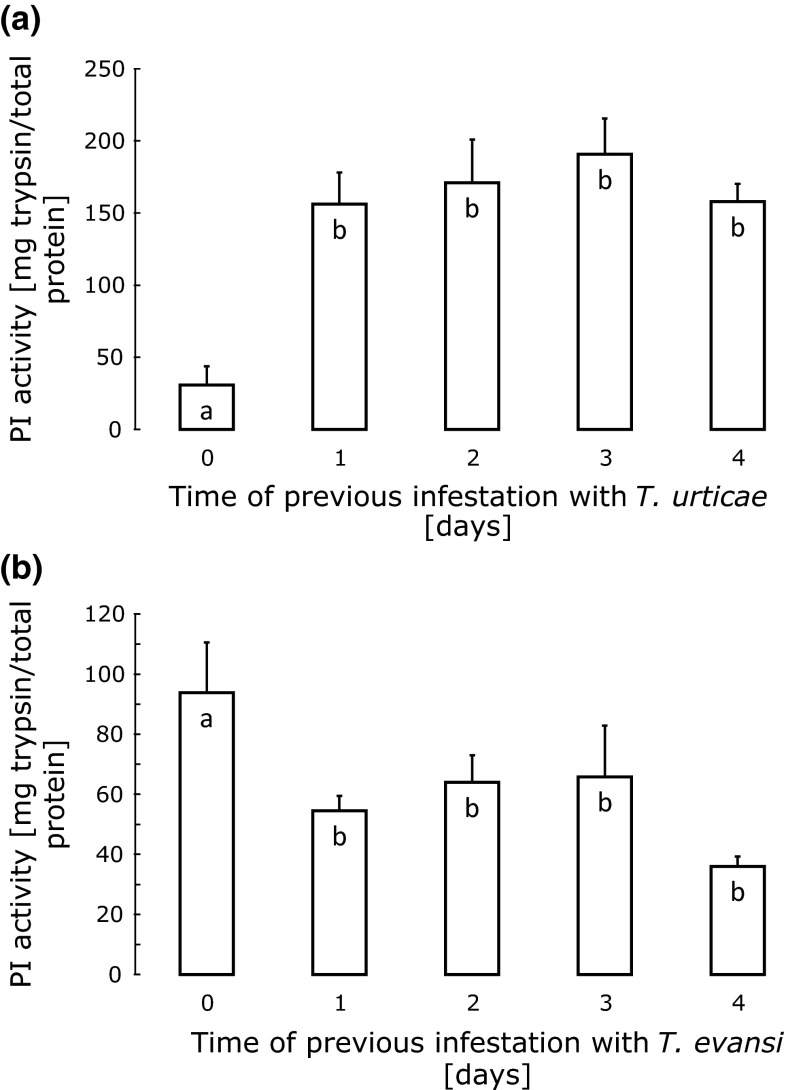
Average proteinase inhibitor (PI) activity (in mg trypsin/total protein + SE) in leaves that were previously attacked by *T. urticae* (**a**) or *T. evansi* (**b**) for 1–4 days, or that were not attacked (0 days). Oviposition rates (Fig. 1) were measured on the same leaves. Within each panel,* bars with the same letter* are not significantly different (contrast among treatments after GLM). See the legend to Fig. 1 for further explanation

There was a significant effect of attacks by both species on proteinase inhibitor (PI) activity in the attacked leaves (Fig. 2a, GLM, *T. urticae*: *F*_4,15_ = 8.6, *P* = 0.0008; *T. evansi*: *F*_4,15_ = 3.19, *P* = 0.044). Levels of PI activity were significantly lower in leaves of unattacked plants (0 days of previous infestation) than in leaves previously attacked by *T. urticae* for 1–4 days (Fig. 2a). In contrast, PI activity was significantly lower in leaves attacked by *T. evansi* than in clean leaves (Fig. 2b). The PI activity showed a negative relation with the oviposition rates: when activity levels were high, oviposition rates were low, and vice versa (cf. Figs 1, 2).

In conclusion, both the oviposition data and the PI activity levels show that the two herbivores affect plant defences within 1 day: whereas *T. urticae* upregulates defences, *T. evansi* downregulates them. We therefore decided to study the effects of simultaneous attack by both species after 1 day of infestation.

### Simultaneous attack by *T. evansi* and *T. urticae*

**Fig. 3:**
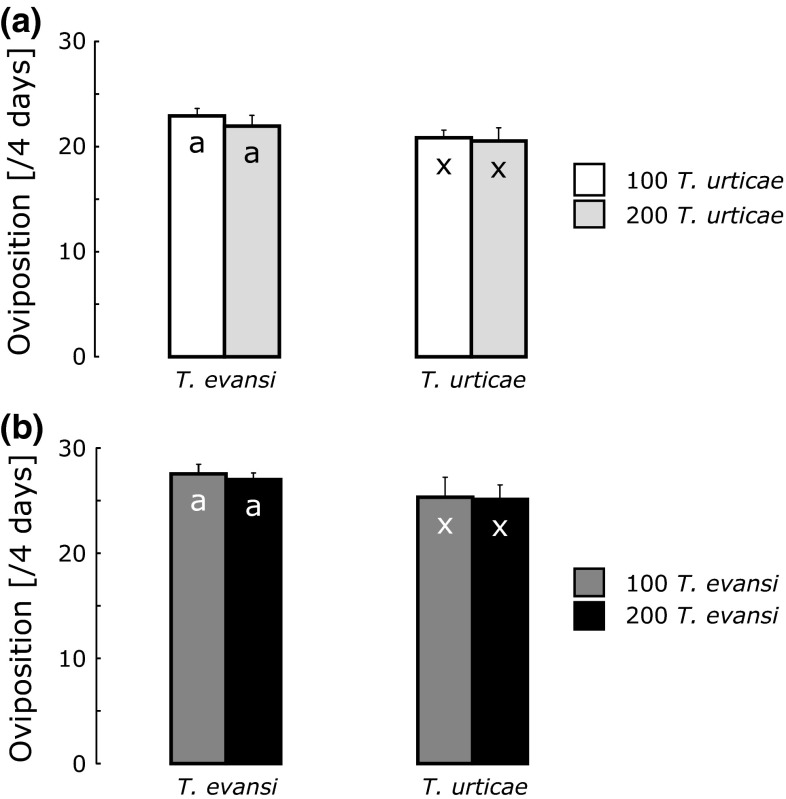
Average oviposition rates (number of eggs per female per 4 days + SE) of *T. evansi* and *T. urticae* on leaves that had previously been infested for 4 days. **a** Previous infestation with 100 (*white bars*) or 200 *T. urticae* (*light gray bars*). There was no effect of the number of mites used for the infestation on the oviposition rate of *T. evansi* (GLM with gamma error distribution: *df* = 1.6, deviance = 0.004, *P* = 0.118) or *T. urticae* (*df* = 1.6, deviance = 0.0005, *P* = 0.769). **b** Previous infestation with 100 (*dark gray bars*) or 200 *T. evansi* (*black bars*). Again, there was no effect of the number of mites on the oviposition of *T. evansi* (*df* = 1.6, deviance = 0.0007, *P* = 0.63) or *T. urticae* (*df* = 1.6, deviance = 0.00014, *P* = 0.93)

The oviposition rates of the two spider mite species did not differ significantly on leaves that were previously attacked by 100 or 200 mites of either species (Fig. 3). We therefore decided to use 100 mites of each species to infest the leaves of the plants, resulting in 200 mites on leaves that were attacked simultaneously and 100 mites on leaves that were attacked by one of the two species.

**Fig. 4:**
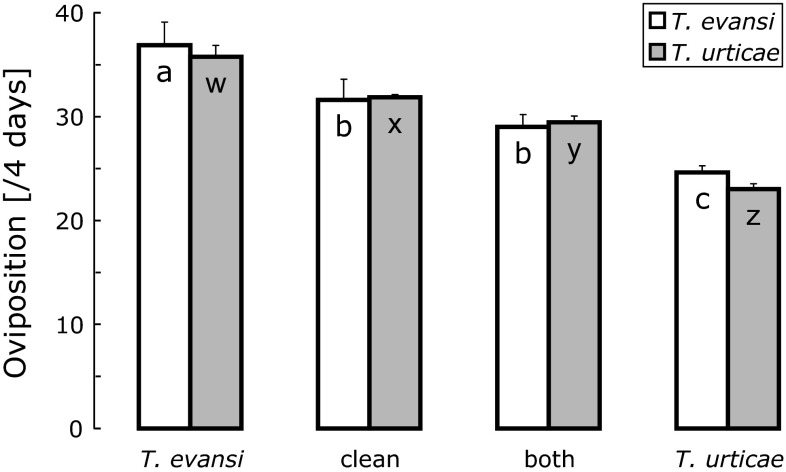
Average oviposition rates (number of eggs per female per 4 days + SE) of *T. evansi* (*white bars*) and *T. urticae* (*gray bars*) on leaves that were previously infested for 1 day by *T. evansi* or *T. urticae*, by both, or were not infested (clean leaves). For each species,* bars with the same letters* do not differ significantly (contrast among treatments after GLM)

The oviposition rates of the two species were significantly affected by the plant treatments (GLM, *T. urticae*: *F*_3,12_ = 61.5, *P* < 0.0001; *T. evansi*: *F*_3,12_ = 9.84, *P* = 0.0015) (Fig. 4). Previous infestation by *T. evansi* for 1 day resulted in higher oviposition rates than on previously uninfested plants for both species, confirming our earlier findings (Sarmento et al. 2011a, b). As expected, a previous infestation by *T. urticae* resulted in lower oviposition rates for both species. Simultaneous infestation resulted in intermediate oviposition rates, which were not significantly different from that on clean plants for *T. evansi*, but it was somewhat lower than that observed on clean plants for *T. urticae* (Fig. 4).

**Fig. 5:**
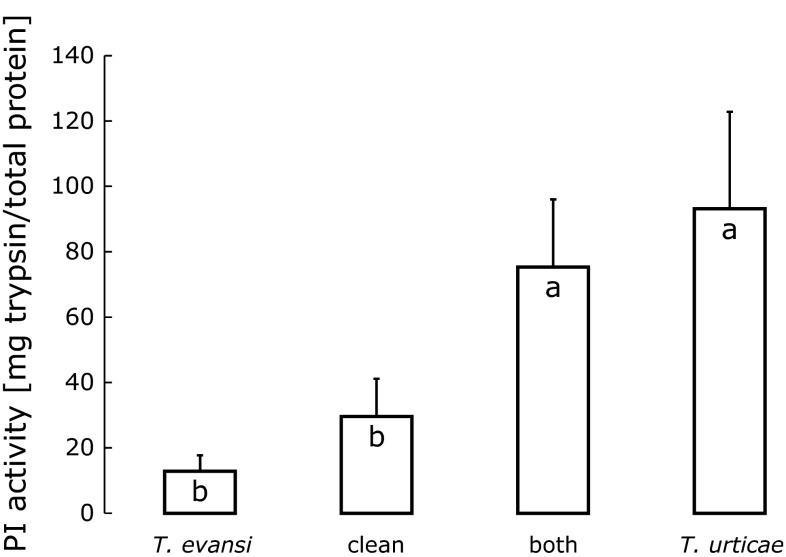
Average proteinase inhibitor (PI) activity (in mg trypsin/total protein, + SE) in leaves previously attacked for 1 day by *T. urticae,* by *T. evansi,* by both, and in uninfested leaves (clean). Oviposition rates (Fig. 5) were measured on the same leaves.* Bars with the same letter* are not significantly different (contrast among treatments after GLM)

PI activity was significantly affected by the infestation (Fig. 5, *F*_3,12_ = 3.87, *P* = 0.038). The activities in leaves previously attacked by *T. evansi* and in clean leaves were significantly lower than in leaves that were previously attacked by *T. urticae* and by both mite species (Fig. 5). Comparison of Figs. 4 and 5 show a less clear negative relation of oviposition to level of PI activity than above (Figs. 1, 2).

## Discussion

Our results confirm earlier findings that *T. evansi* downregulates plant defences (Sarmento et al. 2011a, b; Alba et al. 2015). In particular, both *T. evansi* and *T. urticae* had higher oviposition rates on leaves previously attacked by *T. evansi*. Our results also show that the *T. urticae* used here induces defences in tomato plants (Li et al. 2002; Kant et al. 2004, 2008; Ament et al. 2004), and that *T. evansi* is sensitive to these defences (Sarmento et al. 2011a). The effects of induction as well as reduction of defences on oviposition occurred within 1 day. Indeed, the oviposition rates of the herbivore species on leaves previously attacked by *T. evansi* were highest after 1 day of previous infestation and decreased subsequently (Fig. 1), which could be due to an increase of plant defences because of a longer period of attack and consequently higher damage levels, or because of a decrease in the quality of the leaf discs due to depletion by the herbivores. The fact that the proteinase inhibitor (PI) activity did not increase with the period of infestation by *T. evansi* (Fig. 2b) seems to point to the latter explanation.

Whereas the line of *T. urticae* used here induced plant defences, resulting in lower oviposition rates, *T. evansi* reduced plant defences, causing higher oviposition rates. Surprisingly, the oviposition rates of both species on leaves that were previously attacked by both species simultaneously were intermediate between the oviposition rates observed on leaves previously attacked by either of the two species separately (Fig. 4), suggesting that the effects of both species on the effective plant defences roughly cancel out. Hence, *T. evansi* can reduce plant defences to levels lower than those present in clean plants (Sarmento et al. 2011a) but cannot reduce defences induced by *T. urticae* to those low levels. Likewise, Alba et al. (2015) found that *T. evansi* did not suppress the accumulation of phytohormones involved in plant defence in leaves co-infested with *T. urticae*, but did suppress the expression of downstream defence marker genes. This suggests that the compounds that are possibly involved in the reduction of plant defences by *T. evansi* are not capable of completely circumventing defences, and that elicitors involved in the induction of plant defences by *T. urticae* can partially rescue the defences reduced by such compounds. Possibly, plants cannot cope with these compounds and elicitors simultaneously, but the higher activity of PI observed in leaves attacked by both mites shows that there is at least some defence response in the doubly infested leaves (Fig. 5). This is further confirmed by the oviposition rate of *T. urticae*, which was slightly, but significantly, different on leaf discs from co-infested plants than on leaf discs from clean plants.

The high activity of PI in co-infested leaves (Fig. 5) and the intermediate oviposition rates on these leaves (Fig. 4) suggest that the activity of this defensive compound does not correlate well with the level of plant defences as reflected in herbivore performance. However, the PI levels were measured at the start of the oviposition tests and the activity levels in the leaf discs may have changed during the 4 days of the oviposition assay. We therefore used the oviposition data of the first day of the experiment on simultaneous attack to investigate the correlation between PI activity level and oviposition rates (oviposition data from the experiment on the timing of induction were collected once after 4 days, so they could not be used for this). As with many toxic and defensive compounds, one would expect that low and very low activity levels have no effect on performance, whereas high and very high activity levels would have the maximum effect. We therefore fitted dose–response curves as well as linear models to the data.

**Fig. 6:**
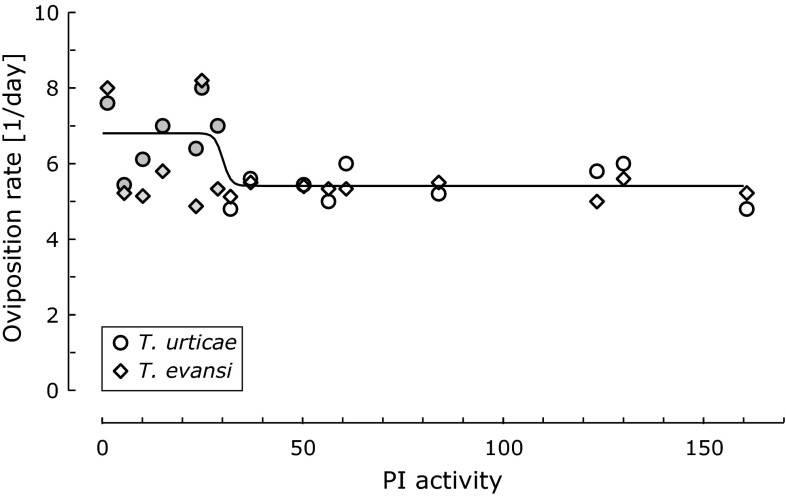
Relationships between proteinase inhibitor (PI) activity (data from Fig. 5) and the oviposition rates (eggs/female/day) of *T. urticae* (*circles*) and *T. evansi* (*diamonds*) on discs from the same leaf (data included in Fig. 4). Proteinase activity levels were assessed at the onset of the oviposition assay; oviposition was measured 1 day later. The seven *gray symbols* correspond to the lowest seven PI activity levels, which were from the four plants previously attacked by *T. evansi* and three of the four clean plants (Fig. 5). The fitted curve is a 3-parameter dose–response curve [Ovip = 6.81 − 1.40/(1 + e^30.08 − *PI*^)] to the oviposition data of *T. urticae*

The correlation between PI activity level and oviposition of *T. urticae* was bordering on significant (Fig. 6,* F*_1,14_ = 4.0, *P* = 0.065). A piecewise regression model did not give a significantly better fit than a linear model, but a three-parameter logistic model gave a significant better fit than the linear model (Fig. 6, *F*_1,13_ = 7.44, *P* = 0.017, AIC of the linear model: 45.3, AIC of the logistic model: 40.1). Neither of the models was significant for *T. evansi*. The seven points of lowest PI activity corresponding to the plateau of high oviposition in *T. urticae* (gray points in Fig. 6) are from the 4 plants that were previously attacked by *T. evansi* and 3 of the clean plants. It is clear that an increase of proteinase inhibitor activity to levels above ~40 does not further decrease the oviposition rate of *T. urticae* (Fig. 6), and that the oviposition rate of *T. evansi* does not correlate with proteinase inhibitor activity. This absence of a linear correlation between PI activity and mite performance shows that it is potentially problematic to use PI activity to quantify the levels of plant defence experienced by the herbivores. This is hardly surprising given the many and varied changes that occur in plants upon attacks by herbivores (Baldwin and Preston 1999; Baldwin et al. 2001; Kant and Baldwin 2007; Alba et al. 2015), but the repercussion of this is that plant defences can only be assessed in a comprehensive way through measurements of herbivore performance because this integrates the impacts of all defensive actions of the plant (Kahl et al. 2000; Consales et al. 2012). Possibly, there is spatial variation in the concentrations of defensive compounds within single leaves (Shroff et al. 2008), and the mites preferentially feed on tissues with low defence levels. In this case, the levels of PI activity measured in this study may not be representative of the tissue on which the mites were feeding. We suggest that it is informative to test other defensive traits for correlation with herbivore performance in a similar fashion.

We show here and elsewhere (Sarmento et al. 2011a) that *T. urticae* can profit from the decrease of plant defences caused by *T. evansi*. Even in doubly infested leaves, *T. urticae* has a higher oviposition rate than on leaves with conspecifics only. In contrast, the performance of *T. evansi* decreases as a consequence of defences induced by *T. urticae*. This suggests that *T. urticae* should preferentially attack plants previously infested by *T. evansi*, and the latter should prefer plants attacked by conspecifics to plants attacked by *T. urticae*. This remains to be tested. Meanwhile, it is clear that the two herbivore species affect each other through induced plant responses, and this can affect the course of within-plant competition between them. However, when populations of the two species were allowed to grow on the same plants, populations of *T. urticae* showed low population growth rates and were outcompeted by *T. evansi*. In contrast, *T. evansi* was not significantly affected by the presence of *T. urticae* (Sarmento et al. 2011b). The profuse web produced by *T. evansi* was probably one of the causes, because it hinders *T. urticae* (Sarmento et al. 2011b), but reproductive interference between the two species may also have played a role (Sato et al. 2014). This shows that assessment of defence-mediated indirect effects among herbivores cannot serve as a prediction for the outcome of competition, and competition experiments are essential to assess the net effect of simultaneous attacks on the population dynamics of the herbivores.

In general, it is thought that chewing insects such as caterpillars induce the jasmonic acid (JA) defence pathway, whereas phloem-sucking insects such as aphids and whiteflies induce the salicylic acid (SA) pathway (Walling 2000, 2008; de Vos et al. 2005; Zarate et al. 2007). However, it is known that several species of spider mites induce both pathways (Kant et al. 2004; Ament et al. 2004; Matsushima et al. 2006), and there is accumulating evidence that *T. evansi* induces neither of the two (Sarmento et al. 2011a, b; Alba et al. 2015). Interactions between these two pathways have often been shown (Thaler et al. 1999, 2002; Arimura et al. 2005; Bruessow et al. 2010). This suggests that herbivores that induce different defensive pathways may increase each other’s performance on co-attacked plants (Rodriguez-Saona et al. 2005, 2010; Bruessow et al. 2010; Soler et al. 2012) because they are differentially susceptible to defences mediated by different signaling pathways that interact with each other (Thaler et al. 2002). We show that the performance of *T. urticae* was improved on plants previously attacked by *T. evansi*, but the opposite was not the case. The increased performance of *T. urticae* shows that positive indirect effects through plant defences are not necessarily restricted to insects with different feeding modes that induce different defensive pathways: *T. urticae* can induce both pathways (Kant et al. 2004) but *T. evansi* appears to induce neither of them (Sarmento et al. 2011a). The two herbivore species studied here are closely related, and both feed on the contents of leaf parenchyma cells. Yet they cause contrasting effects on plant defences and affect each other’s performance on plants through induced defences. In fact, earlier studies have shown considerable variation in the induction of and sensitivity to induced plant defences within one species (Kant et al. 2008). We therefore suggest that it is better to focus on the actual effects of herbivores on plant defences rather than generalizing across feeding modes (Agrawal 2000).

### **Author contribution statement**

EFdO and AJ conceived and designed the experiments. EFdO performed the experiments. AJ and EFdO analyzed the data. AJ, EFdO, and AP wrote the manuscript.
